# Quinacrine Impairs Enterovirus 71 RNA Replication by Preventing Binding of Polypyrimidine-Tract Binding Protein with Internal Ribosome Entry Sites

**DOI:** 10.1371/journal.pone.0052954

**Published:** 2013-01-03

**Authors:** Jianmin Wang, Jiang Du, Zhiqiang Wu, Qi Jin

**Affiliations:** MOH Key Laboratory of Systems Biology of Pathogens, Institute of Pathogen Biology, Chinese Academy of Medical Sciences and Peking Union Medical College, Beijing, People's Republic of China; University of British Columbia, Canada

## Abstract

Since the 1980s, epidemics of enterovirus 71 (EV71) and other enteroviruses have occurred in Asian countries and regions, causing a wide range of human diseases. No effective therapy is available for the treatment of these infections. Internal ribosome entry sites (IRESs) are indispensable for the initiation of translation in enteroviruses. Several cellular factors, as well as the ribosome, are recruited to the conserved IRES during this process. Quinacrine intercalates into the RNA architecture and inhibits RNA transcription and protein synthesis, and a recent study showed that quinacrine inhibited encephalomyocarditis virus and poliovirus IRES-mediated translation *in vitro* without disrupting internal cellular IRES. Here, we report that quinacrine was highly active against EV71, protecting cells from EV71 infection. Replication of viral RNA, expression of viral capsid protein, and production of virus were all strongly inhibited by quinacrine. Interaction of the polypyrimidine tract-binding protein (PTB) with the conserved IRES was prevented by quinacrine. Coxsackieviruses and echovirus were also inhibited by quinacrine in cultured cells. These results indicate that quinacrine may serve as a potential protective agent for use in the treatment of patients with chronic enterovirus infection.

## Introduction

Hand, foot, and mouth disease, which is caused by the Enterovirus genus of the Picornavirus family, is a common viral illness in infants and children. Most hand, foot, and mouth disease infections do not result in serious complications; however, when the pathogen is EV71, the disease can present with serious neurological symptoms such as aseptic meningitis, encephalitis, and acute flaccid paralysis, and may even lead to death [1], [2], [3], [4]. EV71 is a typical Picornaviridae virus with a ∼7.4 kb positive-sense, single-stranded RNA genome. Instead of a 7-methyl guanosine cap, a small viral protein called VPg is linked to the 5′ end of the genomic RNA, and is involved in the initiation of viral RNA genome replication. During infection, picornaviruses, including EV71, initiate translation via direct binding of the ribosome to an internal ribosome entry site (IRES) in the 5′ untranslated region, allowing viral gene expression to occur in a cap-independent manner while host-cell translation is shut down [5]. IRES-mediated translation initiation involves the recruitment of a ribosome to an internal binding site, followed by ribosomal scanning of the mRNA to an appropriate downstream AUG codon [6]. Though some short conserved primary nucleotide sequences may also contribute to the IRES, the secondary and tertiary RNA structure appears to be more important [7], [8]. IRESs are divided into three types based on secondary structure: enteroviruses and rhinoviruses (type I), cardioviruses and aphthoviruses (type II), and hepatitis A virus (type III) [9], [10], [11]. Several cellular factors have been identified as interacting with picornavirus IRESs, including lupus autoantigen (La) and the polypyrimidine-tract binding (PTB) protein that have been shown to stimulate picornavirus translation. La can enhance and correct aberrant poliovirus translation in rabbit reticulocyte lysate [12], [13]. PTB is important for translation initiation mediated by both picornaviral and flaviviral IRES in vivo [14], [15].

Recently, several EV71 outbreaks in Western Pacific Region countries have been observed, including in Malaysia in 1997 [16], Australia in 1999 [2], [17], Singapore in 2000 [18], Japan in 1997 and 2000 [19], [20], and Taiwan in 1998 [21]. EV71 was also confirmed to be responsible for the majority of the 488,955 hand, foot, and mouth disease cases reported in 2008 in China, including 126 fatal cases [22]. Although EV71 has caused extensive damage, no effective vaccines or therapeutic measures are yet available. Additionally, the prevalence of EV71 infections is predicted to increase in the near future [23]. Thus, an effective antiviral therapy against EV 71 infection is urgently needed.

Quinacrine was approved as an anti-malarial drug in the 1930s. It is also used for treating giardiasis [24], [25] and tapeworm infections [26], as well as discoid and subcutaneous lupus erythematosus and inflammation [27], [28], [29], [30], [31]. Quinacrine binds to the prion protein and prevents the formation of prion aggregates *in vitro* and has been tested for the treatment for Creutzfeldt-Jakob disease [32], [33]. Its potential uses as an anti-cancer drug [34], [35] and a non-surgical sterilization method for women have also been studied [36]. In addition to its clinical uses, quinacrine inhibits DNA replication, transcription, and protein synthesis by intercalating into DNA and RNA secondary and tertiary structures, including IRESs [37], [38], [39], [40] suggesting that quinicrine could be used as an anti-viral drug. *In vitro*, quinacrine inhibits IRES-mediated translation of hepatitis C virus [41]. More recently, translation by the IRES elements of encephalomyocarditis virus and poliovirus, but not human p53, were suppressed by quinacrine in a dose-dependent manner [42]. Since quinacrine inhibits translation and infection of many IRES-containing viruses, we hypothesized that it might be able to block infection by EV71 and other enteroviruses. Here we report that both EV71 RNA production and synthesis of viral capsid proteins were strongly inhibited by quinacrine. An in vitro RNA pull-down assay indicated that quinacrine could prevent binding of PTB with EV71 IRES, while overexpression of PTB rescued EV71 replication in the presence of quinacrine. Furthermore, replication of the Coxsakievirus (CoxA10, CoxA16 and CoxB5) and Echovirus (Echo25) were also suppressed by quinacrine. These results indicated that quinacrine and other small intercalating molecules might be used as drugs to treat enterovirus infections.

## Results

### Quinacrine inhibits EV71 replication in cultured cells

**Figure 1:**
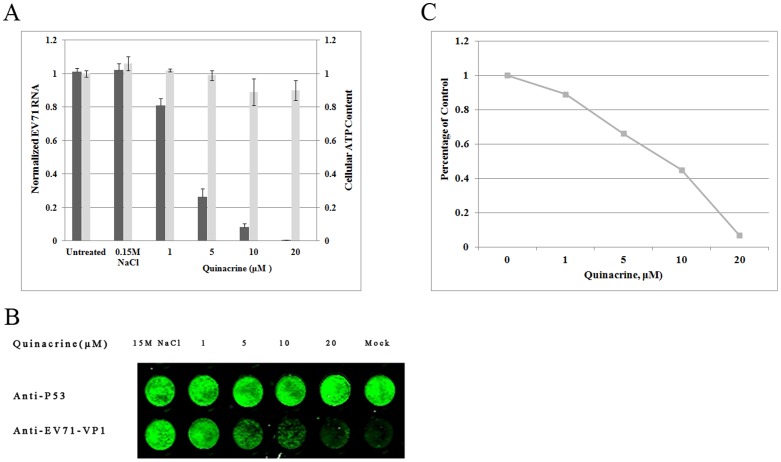
Inhibition of Enterovirus 71 replication by quinacrine. Quinacrine inhibited EV71 RNA accumulation (A), VP1 synthesis (B) and virions produciton in RD cells (*P*<0.05). The experiment was performed in triplicate, and the bars represent means ± SD.

To study the inhibitory effect of quinacrine on EV71, RD cells were infected at 0.1 TCID_50_/cell, and the inhibitory effect on EV71 RNA synthesis was measured at quinacrine concentrations of 1, 5, 10, and 20 µM. EV71 RNA accumulation in infected EV71 cells decreased as the concentration of quinacrine increased (Figure 1A). We found that 5 µM quinacrine inhibited EV71 replication by more than 50%, and almost no copies of EV71 were detected following addition of 20 µM quinacrine. In the infectious cycle, quinacrine inhibited EV71 RNA synthesis in a dose-dependent manner without noticeable cytotoxicity, as measured by cellular ATP content (Figure 1A). The ability of quinacrine to suppress replication of the EV71 RNA genome strongly suggested that overall synthesis of viral proteins and virus would also be inhibited. To test this hypothesis, we infected RD cells with EV71 at 2 MOI in the absence or presence of quinacrine, and used in-cell western blotting to assess the amount of the capsid protein VP1 that accumulated in infected RD cells at 24 hpi; expression of cellular p53 served as the internal control. Synthesis of viral VP1 protein in EV71-infected cells was inhibited by quinacrine in a dose-dependent manner (Figure 1B). When the concentration of quinacrine in the culture medium reached 10 µM, VP1 expression was dramatically reduced, and almost no VP1 expression was detected in the presence of 20 µM quinacrine. This effect was specific to EV71 IRESs, as cellular p53 was not affected by increasing amounts of quinacrine. The culture medium was collected and the yield of infectious EV71 from equal numbers of RD cells was measured three times at 24 hpi by TCID_50_, following the Reed-Muench formula. Production of EV71 virions in the infectious cycle was inhibited by quinacrine in a dose-dependent manner, with an IC_50_ of 9.71 µM/ml (Figure 1C).

### Quinacrine protects cells from EV71 infection

**Figure 2:**
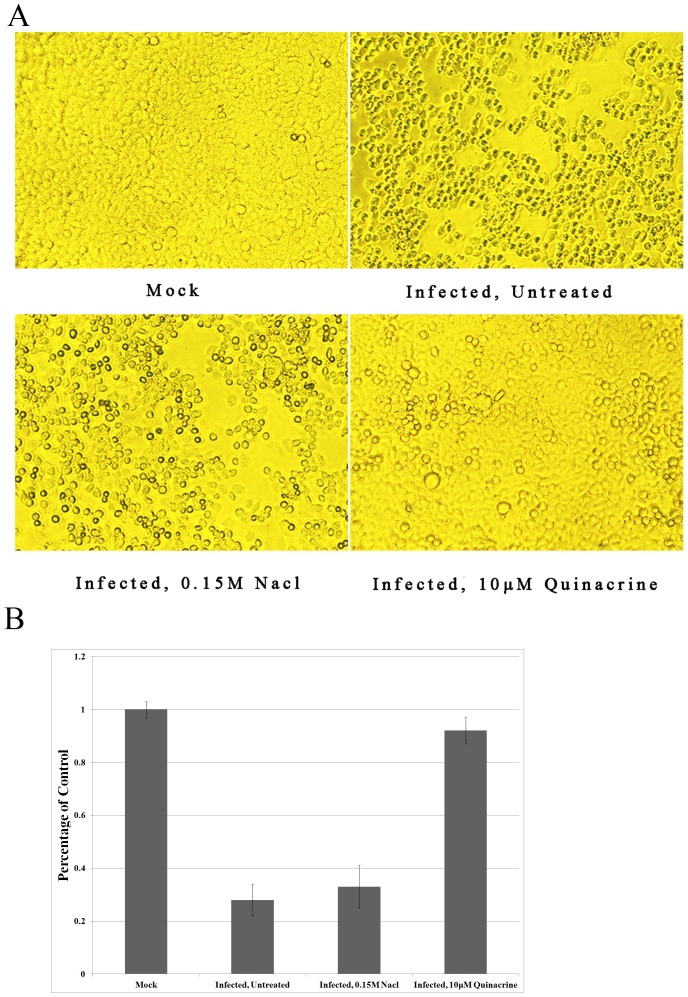
Effects of quinacrine on EV71 infection. (A) Reduction of virus-induced cytopathic effects in RD cells by quinacrine. (B) Quinacrine protected RD cells from EV71 infection (*P*<0.05). The experiment was performed in triplicate, and the bars represent means ± SD.

To study the inhibition of EV71 cytopathic effects (CPE) by quinacrine, RD cells were infected at 0.1 TCID_50_/cell; at the time of infection, 10 µM quinacrine was added to the culture medium. In the negative control experiment, cells were exposed to 0.15M NaCl, which was used to dissolve the quinacrine. Morphological changes in the infected cells were examined by phase-contrast microscopy at 48 hpi. In contrast with the mock-treated control and the negative control, microscopy revealed that the cytopathic effects of EV71 on the RD cells were noticeably inhibited by 10 µM quinacrine (Figure 2A). More than 95% of the mock-treated cells remained alive, while in the negative control and mock-treated group, this number fell to 30% (Figure 2B). After treatment with 10 µM quinacrine, approximately 90% of the EV71-infected cells survived at 48 hpi (Figure 2B).

### A quinacrine-sensitive step after EV71 entry into cells

**Figure 3:**
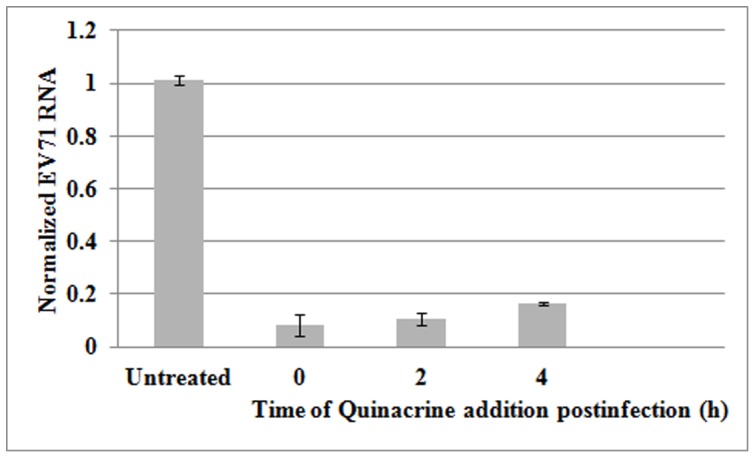
Effects of quinacrine on established EV71 infection. Quinacrine, added 2 or 4 hpi, substantially inhibited EV71 replication in RD cells (*P*<0.05). Standard deviations of three independent experiments are shown.

The inhibitory effect of an anti-viral drug can affect any step in the infectious cycle, including cell entry, protein synthesis, RNA synthesis, or virion assemble and release. To determine whether the inhibitory effect of quinacrine still occurred in an established EV71 infection, quinacrine was added to the culture medium up to 4 hpi. Replication of the EV71 RNA genome was noticeably inhibited by quinacrine even when added at 2 or 4 hpi (Figure 3), and this effect was nearly as strong as when the drug was added at the time of infection, indicating that quinacrine blocked EV71 replication by targeting IRESs after viral entry. Overall, these observations demonstrate that impairment of viral IRES activity by quinacrine limited the effectiveness of EV71's infection of cells.

### Quinacrine blocks PTB binding with the EV71 IRES

**Figure 4:**
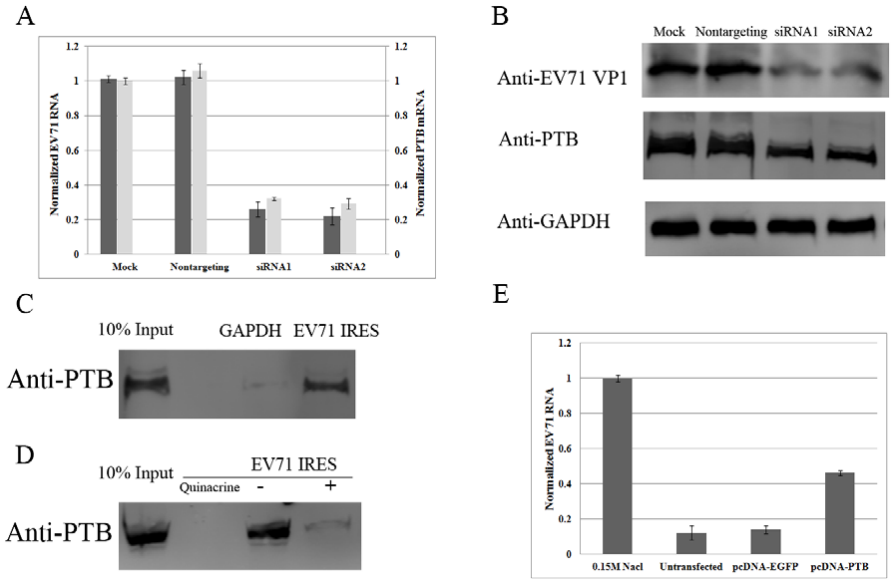
Quinacrine blocks PTB binding with the EV71 IRES in vitro. (A) PTB silencing with two individual siRNA duplexes blocked replication of EV71 (*P*<0.05). Standard deviations of three independent experiments are shown. (B) PTB silencing inhibited expression of EV71 capsid protein. (C, D)RNA pull-down was performed with RD cell lysates, by using biotinylated GAPDH or EV71-IRES transcripts (C) or biotinylated EV71-IRES transcripts with or without quinacrine treatment (D). Western blotting detection was carried with indicated antibodies. (E) PTB overexpression countered viral replication in the presence of quinacrine (*P*<0.05). pcDNA-EGFP and pcDNA-PTB was introduced into RD cells. 24 h later, cells were infected with EV71and treated with quinacrine. Copy numbers of viral genome were determined 12 hpi. Standard deviations of three independent experiments are shown.

Since quinacrine intercalates into the RNA architecture, we hypothesized that it would alter the structure of the IRES and block its binding to cellular factors. We therefore tested the interaction between the EV71 IRES and cellular PTB. To determine whether PTB is required for EV71 replication, PTB was knocked down by siRNA, and replication of EV71 was assayed. In contrast to mock-transfected cells and negative controls, both of the tested siRNAs depleted PTB mRNA transcript levels to around one third of the control (Figure 4A). PTB silencing also strongly inhibited EV71 replication in RD cells (Figure 4A). PTB siRNAs and nontargeting siRNA pretreated RD cells and mock treated RD cells were also infected to test the dependence of viral protein synthesis on cellular PTB expression. Viral protein VP1 and expression of PTB was detected by western blotting. As shown in Figure 4B, while nontargeting siRNA didn't deplete PTB, expression of PTB was reduced to around one third by silencing siRNA duplexes compared with mock control. Expression of viral protein VP1 also remarkably reduced to around 30% compared with mock control and negative control (Figure 4B). In the *in vitro* RNA pull-down assay, PTB was captured by EV71 IRES mRNA but not control GAPDH mRNA, indicating that PTB specifically interacted with the EV71 IRES (Figure 4C). However, after pretreatment with 1 mM quinacrine, the amount of PTB captured by the EV71 IRES was noticeably reduced, suggesting that the ability of the EV71 IRES to bind to PTB was inhibited (Figure 4D). We also tested whether PTB overexpression countered viral replication in the presence of quinacrine. RD cells overexpressing PTB or EGFP as a negative control were infected with EV71 at 0.1 TCID_50_/cell in the presence of 10 µM quinacrine, and the numbers of viral genome copies were determined 12 hpi. Viral genome numbers were substantially higher in cells overexpressing PTB than in EGFP-expressing cells in the presence of quinacrine (Figure 4E).

### Quinacrine inhibits replication of multiple enteroviruses

**Figure 5:**
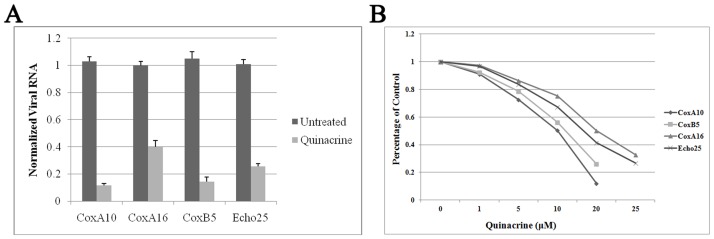
Coxsackieviruses (CoxA10, CoxA16, and CoxB5) and echovirus (Echo25) infection is inhibited by quinacrine. RD cells were infected at 0.1 MOI with various concentrations of quinacrine. (A) Concentrations of 10 µM quinacrine significantly inhibited CoxA10, CoxA16, and CoxB5 and Echo25 replication (*P*<0.05). Standard deviations of three independent experiments are shown. (B) Viral titre was suppressed by quinacrine (*P*<0.05). Standard deviations of three independent experiments are shown.

Since the involvement of cellular PTB is shared by all enterovirus IRESs, we determined the effectiveness of quinacrine against four other enteroviruses: three coxsackieviruses (CoxA10, CoxA16, and CoxB5) and echovirus (Echo25). RD cells were infected with 0.1 TCID_50_/cell and treated with quinacrine in a concentration series at the time of infection. Reverse transcription PCR and relative quantitative real-time PCR were performed to measure the inhibitory effect of quinacrine on the replication of these enteroviruses 24 hpi. The coxsackieviruses and echovirus tested here were all sensitive to quinacrine treatment; replication of all four viruses was inhibited by more than 50%, with inhibition of CoxA10 and CoxB5 as high as 80%, nearly the efficacy of quinacrine against EV71 (Figure 5A). Infectious virions yield was also determined. As shown in Figure 5B, all four viruses were noticeably inhibited by quinacrine in a dose-dependent manner.

## Discussion

To date, several clinical therapies against EV71 infection have been explored. Ribavirin [43], brefeldin A [44]and aurintricarboxylic acid [45] were reported to inhibit EV71 replication *in vitro*. siRNAs against EV71 infection is being researched [46]. However, none of these drugs are currently clinically available. Using high-throughput translation screening, several nucleic acid-binding agents were identified as protein synthesis inhibitors [41], [47], opening new avenues for developing more potent agents against RNA viruses that are based on unique viral RNA sequence and folding. Recently, Gasparian *et al.* reported that quinacrine suppresses IRES-dependent translation initiation without disrupting the cellular p53 IRES and effectively reduces viral protein production as early as 3 hpi [42]. In the current study, the targeting of IRES by quinacrine was highly effective against infectious EV71 in cell cultures, even at relative low concentrations and without obvious cytotoxicity. The life cycle of picornaviruses is rapid; virus entry, uncoating, and translation generally occur within 2 hpi, while viral RNA replication is initiated around 3 hpi. Here, we observed a quinacrine-sensitive step after EV71 entry into cells. IRES-directed quinacrine specifically reduced viral replication whether applied to cultured cells at the time of infection or four hours later.

PTB has been shown to interact with EMCV, foot-and-mouth disease virus, human rhinovirus, and poliovirus IRESs, and is important for translation initiation mediated by the EMCV IRES [48], [49], [50]. In our investigation, PTB depletion by siRNA led to suppression of EV71 replication in cultured cells. PTB was also captured by the EV71 IRES *in vitro*, reflecting the interaction between EV71 and cellular PTB protein. A further experiment showed that this interaction was altered by quinacrine, which accounted for its inhibitory effect on EV71 replication. Overexpression of PTB countered EV71 replication in the presence of quinacrine, which was consistent with the previous observation that overexpression of PTB leads to an increase in IRES activity [15]. This result suggests that PTB may act as a chaperone to stabilize IRES structure by avoiding intercalation of quinacrine and by maintaining an IRES conformation suitable for its recognition by the translational machinery. Importantly, in this study quinacrine inhibited not only EV71 but also clinical isolates of coxsackieviruses CoxA10, CoxA16, and CoxB5, as well as echovirus Echo25.

In conclusion, we have demonstrated that quinacrine exerts a strong inhibitory effect on EV71 replication. This finding is particularly important because no effective antiviral drug is currently available for the prevention, treatment, and control of fatal EV71 infections in humans. Quinacrine has a long history of clinical use to treat protozoal infections and rheumatic diseases, which suggests its safety. Overall, results from this study show that quinacrine has therapeutic potential against a broad spectrum of human enteroviruses, including EV71.

## Materials and Methods

### Cell culture and drug treatment

Rhabdomyosarcoma (RD, ATCC, USA) cells were propagated and maintained in minimum essential medium (HyClone, Logan, USA) supplemented with 10% fetal bovine serum (Invitrogen, Carlsbad, USA) and 100 U/ml penicillin, and 100 µg/ml streptomycin at 37°C with 5% CO_2_. Quinacrine (Sigma-Aldrich, St. Louis, USA) was dissolved in 0.15 M NaCl and stored at a concentration of 10 mM at −20°C before use. Cell numbers and proliferation were determined by direct cell number counting using CountStar (Inno-Alliance Biotech, Beijing, China) after stained by trypan blue.

### Virus isolation and infection

The EV71 strain (Shzh-98, GenBank accession no. AF302996) was used. Enteroviruses, CoxA10, CoxA16, CoxB5 and Echo25, were isolated from patients with clinically diagnosed infections. Viruses were propagated in RD cells and infected at a 0.1–2 multiplicity of infection (MOI) per cell, measured as 50% tissue culture infectious doses (TCID_50_). Quinacrine (Sigma-Aldrich, St. Louis, USA) was added to cell cultures in a concentration series at the time of infection or at the indicated times postinfection. In-cell western blotting and quantitative real-time PCR were performed 24 hours postinfection (hpi) as described below.

### Ethics statement

All the patients with clinically diagnosed infections were informed and the written informed consents were acquired before any samples were colected. This procedure was approved by the ethics committee of Institute of Pathogen Biology.

### Viral titer assay

Virus titer in supernatants was determined as TCID_50_ on RD cells by the Reed-Muench method [51]. Representative results are shown. The 50% inhibitory concentration (IC_50_) of quinacrine was calculated using the Forecast function of Microsoft Excel.

### Quantitative Real-Time PCR

At 24 hpi, total cellular RNA and viral RNA were extracted from each well by using the RNAeasy Mini kit (Qiagen, Hilden, Germany) according to the manufacturer's instructions, and reverse transcribed using Superscript First-Strand Synthesis System (Invitrogen, Carlsbad, USA) in a 20 µl reaction mixture with 1.2 µg total RNA according to the manufacturer's protocol. Real-time PCR was conducted using an ABI Prism 7000 Real-time PCR System (Applied Biosystems, Carlsbad, USA) and a Power SYBR Green PCR Master Kit (Invitrogen, Carlsbad, USA). Reactions contained 2 µl of cDNA, 1 µl of each primer and 25 µl Power SYBR Green PCR Master Mix in total volume of 50 µl. Efficiency-corrected relative quantitation was used with glyceraldehyde 3-phosphatase dehydrogenase (GAPDH) as an internal control [52]. The following primers were used: for enteroviruses, primers qEV-F (5′-CCCCTGAATGCGGCTAAT-3′) and qEV-R (5′-CAATTGTCACCATAAGCAGCCA-3′); for GAPDH, primers ( (5′-CTCTGCTCCTCCTGTTCGAC-3′) and qGAPDH-R (5′-TTAAAAGCAGCCCTGGTGAC-3′).

### In-cell western blot analysis

In-cell western blot analyses were performed as described previously [53]. Cells were plated in 96-well plates at 6×10^4^ cells per well and infected the next day with EV71 at 2 MOI. Quinacrine was added to the medium at the time of infection. At 24 hpi, cells were fixed with 4% paraformaldehyde for 30 min and permeabilized with 0.5% Triton X-100 for 15 min. Cells were washed twice with PBS and incubated with anti-EV71 VP1 monoclonal antibody (eENZYME, Maryland, USA) and mouse anti-p53 (Beyotime, Suzhou, China) overnight at 4°C. The next day, cells were washed with 0.1% Tween-20 in PBS and incubated with goat anti-mouse 680 (1∶500) (Li-Cor, Lincoln, USA). After washed twice with PBST and twice with PBS, cells were scanned using an Odyssey Infrared Imager (Li-Cor Inc., Lincoln, USA).

### Western blotting

Cells were collected and washed with PBS twice before lysing in buffer containing 100 mM NaCl, 20 mM Tris (pH 8.0), 0.5% NP-40, 0.25% sodium deoxycholate, 1 mM EDTA with proteinase inhibitor cocktail. Supernatant was collected at 13,000 rpm/min for 15 min and resolved by electrophoresis in denaturing 4 to 10% SDS-PAGE and transferred to nylon polyvinylidene difluoride (PVDF) membranes (Hybond P, Piscataway, USA). Membranes were blocked with 5% nonfat dry milk and probed with primary antibodies as indicated at 4°C overnight, followed by incubation with the corresponding IRD Fluor 680-labeled IgG secondary antibody (Li-Cor Inc., Lincoln, USA). After washing, membranes were scanned using an Odyssey Infrared Imaging System (Li-Cor, Lincoln, USA) at the recommended wavelength and analyzed with Odyssey software. EV71 capsid protein VP1was detected by anti-EV71 VP1 monoclonal antibody (eENZYME, Maryland, USA) as follows. Cellular PTB protein was detected by anti-PTB monoclonal antibody (Invitrogen, Carlsbad, USA). Molecular sizes of proteins were determined by comparison with prestained protein markers (Fermentas, Maryland, USA). To control for protein loading, levels of housekeeping protein GAPDH were assessed using mouse anti-GAPDH (Beyotime, Suzhou, China) and IRD Fluor 680-labeled IgG secondary antibody (Li-Cor Inc., Lincoln, USA).

### siRNA design and transfection

siRNAs against PTB were designed and custom synthesized by Invitrogen with the following sequences: siRNA-1, 5′-GGCAGGAAATTCTGTATTG-3′; siRNA-2, 5′-GGAAATGACAGCAAGAAG-3′. Stealth RNAi siRNA Negative Control Med GC (Invitrogen) was used as a negative control. siRNA was introduced into RD cells by transfection using Oligofectamine Reagent (Invitrogen, Carlsbad, USA) according to the manufacturer's instructions. Cells were cultured overnight to 40% confluence and 100 pmol siRNA was added in to 60 µl Opti-MEM® (Invitrogen, Carlsbad, USA) for 15 min at room temperature (RT), while 5 µl Oligofectamine was incubated with 15 µl Opti-MEM. The siRNA and Oligofectamine was mixed and incubated for 20 min at RT before adding to cell cultures. The culture medium was changed after 4 hours and cells cultured for 72 hours before virus infection.

### Plasmid Construction

For transient expression in RD cells, cellular PTB was cloned into pcDNA3 (Invitrogen, Carlsbad, USA) under the control of the cytomegalovirus promoter. The primers used for amplification were 5′- ATGGACGGCATTGTCCCAGA-3′ (forward) and 5′- CTAGATGGTGGACTTGGAGA-3′ (reverse) for constructing pcDNA3-PTB. A FLAG-tag (DYKDDDDK) was anchored to the N-terminus for detection via western blotting. The sequences of the plasmids and the orientation of the inserted fragments were verified by sequencing.

### Synthesis of biotinylated transcripts and RNA pull-down assay

For *in vitro* synthesis of biotinylated transcripts, total RNA was reverse transcribed using the Superscript First-Strand Synthesis System (Invitrogen) and used as template for PCR with 5′ oligonucleotides that contained the T7 RNA polymerase promoter sequence (5′-CCAAGCTTCTAATACGACTCACTATAGGGAGA-3′). The primers 5′-(T7) TTAAAACAGCTGTGGGTTGTCA-3′ and 5′-CCCATGGTTTTGCTGTGTTGA-3′ were the forward and reverse primers, respectively, for amplification of the EV71 IRES. As a control, cellular GAPDH was amplified with the forward and reverse primers 5′-(T7) CCTCAACGACCACTTTGTCA-3′ and 5′-GGTTGAGCACAGGGTACTTTATT-3′, respectively. The PCR-amplified products were resolved on agarose gels, purified, and used as template for synthesis of the corresponding biotinylated RNAs using T7 RNA polymerase (Promega, Fitchburg, USA) and biotin RNA Labeling Mix (Roche, Basel, Switzerland) according to the manufacturers' instructions. Three micrograms of biotinylated RNA were heated to 90°C for 2 min, then slowly cooled to RT to allow proper folding of secondary structures. Folded RNA was then incubated with cell lysate for 1 h at RT with continuous stirring to perform biotin pull-down assays. Complexes were isolated using streptavidin-conjugated Dynabeads (Invitrogen), and bound proteins in the pull-down material were analyzed by western blotting. In the inhibition assay, folded RNA was incubated with 1 mM quinacrine for 2 h at room temperature before addition to the cell lysate.
